# A Small Calculus Removed by the Lateral Operation of Lithotomy

**Published:** 1855-08

**Authors:** Paul F. Eve

**Affiliations:** Professor of Surgery, Med. Dep. Uni. of Nashville


### RECORD OF MEDICAL SCIENCE.

*A Small Calculus Removed by the Lateral Operation of Lithotomy.* By PAUL F. EVE, M. D., Professor of Surgery, Med. Dep. Uni. of Nashville.—From the statistical report of the principal operations performed in the London hospitals during the month of January, 1855, we find the following case re-published in the Medical News of Philadelphia: "A healthy child, aged 4, under the care of Mr. Athol Johnson, in the hospital of children. There had always been great difficulty in detecting the stone. At the operation it was found to be too small to be grasped by the forceps, and was finally removed by the scoop-end of a director and the finger. It weighed only *five grains.* The child recovered well."

My friend, Prof. Wm. H. Van Buren, of New York city, reported in the Medical Times for May, 1853, the case of a child 3 years old, who from birth labored under symptoms of stone. Some difficulty was experienced in detecting the calculus, but by the lateral operation, one weighing *nine grains* was successfully extracted.

"Mr. Martineau, of Norwich, England, is said by Crosse in his prize essay on Urinary Calculus, to have extracted a calculus weighing only *ten grains* from a boy 13 years old; and this is spoken of as being the *smallest probably ever removed by operation.*"

In the 1000 extracted by the late distinguished Dr. Physick from Chief Justice Marshall, of Virginia, some, of course, were represented as exceedingly diminutive—comparable to pin-heads. In my own collection of 157, the smallest single calculus weighed *eleven grains;* but when multiple, some weighed only a grain or two.

I recollect a case, some years ago, in which I was called by the attending physician to relieve difficult micturition. As the catheter entered the bladder, I felt pretty certain it had encountered a rough foreign body at its neck. The patient was now prepared for lithotrity, as he had an ample urethra and good condition of the bladder for this operation. But by no manipulation with instruments, could a calculus be again detected. A few days afterwards, he brought me a stone, about the size of a small grain of coffee, which he had passed in urinating.

The case now reported is also that of a boy, 4 years of age, who had experienced symptoms of stone in the bladder for the past ten or twelve months. Its existence was detected by the sound about four months before the operation.

The child being evidently of a scrofulous habit, and having, too, recently passed worms, was put under treatment; and at the end of ten weeks was supposed to be prepared for lithotomy. Placed under the influence of a combination of ether and chloroform, no one now present could be certain of touching the calculus, and the operation was deferred.

Two months later, April 21st, the distressing symptoms of a foreign body in the bladder still persisting, preparations were again made for its removal. As the grooved-staff entered this organ, it apparently pushed the stone from its neck; but by no subsequent manipulation, either with it in my hand or in that of others, could a *click, clink,* or sonorous sound be elicited. Satisfied that I had again touched a rough foreign body, and which was confirmed by Dr. Briggs, we proceeded to perform the bi-lateral operation. As even the smallest size *double lithotome caché* of Dupuytren was deemed too large for our little patient, one manufactured here for the purpose was employed. The blades were, however, found too slender to divide the parts with facility and certainty, when this instrument was laid aside, and the lateral section made with a scalpel. A very small calculus was now detected in the bladder, which could not be seized with forceps, but was readily extracted with a finger *It weighed eight grains.* The child had a very rapid recovery. In three or four days he was about the house.

In these instances there was not substance or material enough to produce the metallic ring or click when the sound touched the urinary concretion. This diagnostic symptom, the most pathognomic of vesical calculus, is absent in these cases, and the operation must therefore be performed under peculiar circumstances. Neither can the finger in the rectum, on account of the small size of the stone, add much or anything in making out definitely the correct diagnosis. The surgeon has chiefly to rely upon the *tactus eruditus* in these cases.—*Nashville Jour. Med.*

